# Urokinase receptor and CXCR4 are regulated by common microRNAs in leukaemia cells

**DOI:** 10.1111/jcmm.12617

**Published:** 2015-06-16

**Authors:** Daniela Alfano, Anna Gorrasi, Anna Li Santi, Patrizia Ricci, Nunzia Montuori, Carmine Selleri, Pia Ragno

**Affiliations:** a Department of Chemistry and Biology, University of SalernoSalerno, Italy; b Department of Clinical Medicine and Surgery, “Federico II” UniversityNaples, Italy; c Department of Translational Medical Sciences, “Federico II” UniversityNaples, Italy; d Department of Medicine and Surgery, University of SalernoSalerno, Italy

**Keywords:** urokinase receptor, uPAR, CXCR4, MicroRNA, AML

## Abstract

The urokinase-type plasminogen activator (uPA) receptor (uPAR) focuses uPA proteolytic activity on the cell membrane, promoting localized degradation of extracellular matrix (ECM), and binds vitronectin (VN), mediating cell adhesion to the ECM. uPAR-bound uPA and VN induce proteolysis-independent intracellular signalling, regulating cell adhesion, migration, survival and proliferation. uPAR cross-talks with CXCR4, the receptor for the stroma-derived factor 1 chemokine. CXCR4 is crucial in the trafficking of hematopoietic stem cells from/to the bone marrow, which involves also uPAR. Both uPAR and CXCR4 are expressed in acute myeloid leukaemia (AML), with a lower expression in undifferentiated and myeloid subsets, and higher expression in myelomonocytic and promyelocytic subsets. We hypothesized a microRNA (miR)-mediated co-regulation of uPAR and CXCR4 expression, which could allow their cross-talk at the cell surface. We identified three miRs, miR-146a, miR-335 and miR-622, regulating the expression of both uPAR and CXCR4 in AML cell lines. Indeed, these miRs directly target the 3′untranslated region of both uPAR- and CXCR4-mRNAs; accordingly, uPAR/CXCR4 expression is reduced by their overexpression in AML cells and increased by their specific inhibitors. Overexpression of all three miRs impairs migration, invasion and proliferation of myelomonocytic cells. Interestingly, we observed an inverse relationship between uPAR/CXCR4 expression and miR-146a and miR-335 levels in AML blasts, suggesting their possible role in the regulation of uPAR/CXCR4 expression also *in vivo*.

## Introduction

The receptor (uPAR) for the urokinase-type plasminogen activator (uPA) is a three-domain receptor, anchored to the cell-surface by a GPI tail. uPAR focuses uPA proteolytic activity on the cell membrane, promoting localized extracellular matrix (ECM) degradation, and, at the same time, mediates cell adhesion to vitronectin (VN), an ECM component abundant in tumour tissues 1. uPAR, despite its GPI-tail, is able to activate cell-signalling pathways by associating to cell-surface molecules, in particular to integrins and to the chemotaxis receptors for the formylated peptide f-Met-Leu-Phe (fMLF-Rs) 2,3. uPA, VN and uPAR overexpression itself induce proteolysis-independent intracellular signalling, regulating cell adhesion, migration, survival, proliferation and pericellular proteolysis 4–6. uPAR can be cleaved and/or shed from the cell surface, thus generating cleaved and soluble forms. A soluble form of cleaved uPAR (DIIDIII-suPAR), lacking the N-terminal domain and exposing the SRSRY sequence (aa 88-92), binds and activates the fMLF-Rs, thus inducing directional cell migration 7,8. Both cell-surface and DII-DIII-suPAR are able to regulate the activity of CXCR4, the receptor of the stroma-derived factor 1 (SDF1) chemokine, by a fMLF-R-dependent mechanism 9,10.

CXCR4 is implicated in several physiological and pathological processes; in fact, it is a co-receptor for T-trophic HIV, plays a fundamental role in foetal development and in the trafficking of naıve lymphocytes and is a key molecule in the regulation of hematopoietic stem cell (HSC) trafficking from and to the bone marrow (BM) 11. Hematopoietic stem cells are characterized by expression of CD34 antigen and negativity of lineage markers. The majority of CD34^+^ HSCs reside in the BM, only few circulating in peripheral blood (PB). CXCR4 strongly contributes to HSC retention in BM since it is highly expressed in HSCs, and its ligand, SDF1, is largely produced by BM endothelium; in fact, AMD3100, a CXCR4 antagonist, is a potent HSC mobilizer 12.

Previously, we reported uPAR involvement in HSC mobilization 10,13. In fact, administration of granulocyte colony-stimulating factor, the most common HSC mobilizer, to HSC healthy donors, increased serum DIIDIII-suPAR levels, which likely contributed to HSC mobilization by directly inducing migration of BM-HSCs into the circulation and/or inactivating their CXCR4 10. By contrast, the soluble form of full length uPAR (suPAR), increases HSPCs chemotactic response to SDF1 14. Interestingly, in mice, the membrane-anchored uPAR marks a subset of BM-HSPCs and is released during mobilization; uPAR loss impairs HSPC homing and engraftment to the BM microenvironment 15. uPAR also mediates mobilization, migration and differentiation of mesenchymal stem cells 16.

Biochemical mechanisms regulating mobilization into PB and homing and engraftment to BM of normal HSCs and leukaemia cells are likely similar 17; recent evidence suggest that leukaemic cells share surface molecules in common with stem cells and may be mobilized under similar conditions 18.

Both uPAR and CXCR4 expression is strongly up-regulated and represents a negative prognostic factor in various cancers, including haematological malignancies 11,19,20. Among lymphoproliferative disorders, uPAR expression is exclusively found in pathological plasma cells. High levels of soluble uPAR appear to represent an independent factor predicting worse prognosis and extramedullary involvement in multiple myeloma 21. In acute myeloid leukaemia (AML), a high uPAR expression associates to a greater tendency to cutaneous and tissue infiltration 21,22. CXCR4 is also expressed in myeloid and lymphoid leukaemia cells, with a major prognostic impact in AML 11,19. *In vitro* and *in vivo* evidence suggest that CXCR4 expression by leukaemia cells allows for their homing and retention within the BM, accessing niches that are normally restricted to progenitor cells. CXCR4- and integrin-mediated contact between leukaemia cells and stromal cells protects them from spontaneous and chemotherapy-induced cell death 23,24.

Both uPAR and CXCR4 are differentially expressed in AML, with lower expression in undifferentiated (M0), myeloid (M1/2) and erythroid (M6) AML, and higher expression in promyelocytic (M3) and myelomonocytic (M4/5) AML 22,25.

uPAR and CXCR4 expression can be regulated by various factors, both at transcriptional and post-transcriptional levels 1,11,26. Key players in the post-transcriptional regulation of gene expression are small non-coding RNAs, termed microRNAs (miRs). MiRs are regulatory single-strand RNAs that typically consist of 20–23 nucleotides in length; they regulate gene expression by pairing with target mRNAs, thus inhibiting their translation and, often, inducing their degradation 27,28. MiRs play key roles in many biological processes.

MiR expression changes dynamically during hematopoiesis; in fact, miRs control differentiation and activity of hematopoietic cells by targeting transcription factors, growth factor receptors and molecules involved in the modulation of cellular responses to external stimuli 29,30. MiRs are frequently deregulated in human malignancies and have shown great potential as biomarkers for diagnosis and prognosis and as target in therapy 31,32. Distinctive patterns of increased expression and/or silencing of multiple miRs (miR signatures) have been observed in AML and have been associated with specific cytogenetic and molecular subsets of AML 33–35. MiR-mediated regulation of uPAR or CXCR4 expression has been scarcely investigated.

In summary, HSC mobilization is associated to down-regulation of uPAR and CXCR4 expression/activity on their surface and, viceversa, HSC homing and engraftment to BM require expression of CXCR4 and, at least in mice, of cell-surface uPAR. Both receptors are regulated in the same direction in AML subsets and, further, cross-talk at the cell-surface. MiRs are multitarget molecules involved in haematopoiesis and deregulated in AML.

On these basis, we hypothesized that uPAR and CXCR4 expression could be co-regulated by same miRs in AML, regulating AML cell functions. We identified three miRs targeting both uPAR and CXCR4; identified miRs were validated and their expression and functions were examined in leukaemia cell lines and in blasts from AML patients.

## Materials and methods

### Reagents

The R2 anti-uPAR monoclonal antibody was kindly provided by G. Hoyer-Hansen (Finsen Institute, Copenhagen, Denmark). Rabbit poyclonal anti-CXCR4 antibody was from Upstate (Temecula, CA, USA). Rabbit anti-actin, mouse anti-tubulin antibodies, the protease inhibitor cocktail and Collagen VI were from Sigma-Aldrich (St. Louis, MO, USA). pGL3 vector, pRLSV40 plasmid and dual-luciferase reporter assay system were from Promega (Madison, WI, USA). Lipofectamine 2000 and Oligofectamine transfection reagents were purchased from Invitrogen (Paisley, UK). The Nucleofector kit was from Lonza (Basel, Switzerland). Pre-miRs were from Ambion (Austin, TX, USA). Mercury LNA inhibitors were from Exiqon (Vedbaek, Denmark). Lymphoprep was from Stem cell Technologies (Vancouver, BC, Canada); anti-CD3 Abs and IgG-conjugated magnetic beads for immunodepletion were from Life Technologies (Carlsbad, CA, USA). Horseradish peroxidase-conjugated anti-mouse and anti-rabbit IgG and IQ™SYBR Green Supermix were from Bio-Rad (Hercules, CA, USA). ECL (Enhanced ChemiLuminescence) detection kit was from Amersham International (Amersham, UK) and polyvinylidene fluoride (PVDF) filters from Millipore (Windsor, MA, USA). The chemotaxis polyvinylpyrrolidone-free (PVPF) filters from Whatman Int. (Kent, UK). QuantiTect Reverse Transcription kit was from Qiagen (Hilden, Germany). MicroRNA Assay kit and Qiazol reagent were from Life Technologies (Carlsbad, CA, USA).

### Patient specimen collection

Bone marrow samples were obtained, after informed consent, during diagnostic procedures from 10 AML patients (FAB classification: 1M1, 3M2, 1M3, 4M4, 1M5). Diagnosis was based on MGG-stained BM smears, cytochemistry and immunophenotyping. No patient had a history of prior therapy with anticancer drugs or a preceding diagnosis of myelodysplastic syndrome.

Mononuclear cells were isolated by density gradient centrifugation using Lymphoprep. Samples with less than 80% blasts were depleted from contaminating T cells using antibodies and magnetic beads, as described previously 36, resulting in a final blast purity ≥95% as determined by morphology on cytospin preparations. Cells were lysed in TRIzol Reagent and total RNA extracted according to the manufacturer’s instructions.

### Cell culture

KG1 acute myelogenous, THP-1 and U937 promonocytic leukaemia cell lines were cultured in RPMI 1640 supplemented with 10% heat-inactivated foetal bovine serum (FCS). Cervical carcinoma Hela cells and prostate carcinoma PC3 cells were cultured in DMEM supplemented with 10% FCS.

### *In vitro* transfection with synthetic miRs or antagomiRs

3 × 10^5^ PC3 or 2 × 10^5^ HeLa cells or 8 × 10^5^ THP-1 cells were plated in 35 mm plates and, after 24 hrs, transiently transfected with precursors of selected or control miRs (40 nM) in antibiotic/serum free medium, using Oligofectamine, according to the manufacturer’s instructions. Cells were lysed after indicated times for Western blot analysis.

For transfection with antagomiRs, 2 × 10^6^ KG1 cells were transfected with 200 nM LNA-ON inhibitors using the Amaxa cell line Nucleofector kit R, according to the manufacturer’s instructions. Cells were lysed after 24 hrs for Western blot analysis.

### Construction of reporter plasmids

A 319-bp fragment encompassing uPAR 3′UTR and a 520-bp fragment encompassing CXCR4 3′UTR (http://genome.ucsc.edu/) were amplified by PCR from human genomic DNA utilizing, for uPAR, the following sense (5′-GCTCTAGAACCTGAAATCCCCCTCTCTGCC-3′) and antisense (5′-GCTCTAGACCACTGGTACAAAATCTTTATGTAAG-3′) primers and, for CXCR4, the following sense (5′-GCTCTAGACACAGATGTAAAAGAC-3′) and antisense (5′-GCTCTAGAATTCAAATTGTACATG -3′) primers, adapted to the XbaI site, using standard procedures and a proofreading polymerase (Platinum *Pfu*; Invitrogen). Each PCR product was individually inserted into the pGL3 vector, using the XbaI site immediately downstream the stop codon of *firefly-*luciferase reporter gene, thus obtaining the pGL3-3′UTR/uPAR or the pGL3-3′UTR/CXCR4 construct. Both constructs were checked by sequence analysis.

### Luciferase assay

HeLa recipient cells were grown into 24-well plates (7 × 10^4^/well) for 24 hrs. Then, cells were transiently co-transfected with 150 ng of pGL3-3′UTR/uPAR or pGL3-3′UTR/CXCR4 constructs, 5 ng of pRLSV40 reporter plasmids containing the *Renilla*-luciferase for normalization, and with 5 pmol of precursors of selected or control miRs. Cells were transfected by Lipofectamine 2000, according to the manufacturer’s instructions. After 24 hrs, transfected cells were lysed and the luciferase activity was measured with a luminometer using the dual-luciferase reporter assay system, according to the manufacturer’s instructions.

### Western blot analysis

Cells were lysed in 1% Triton X-100 in the presence of protease inhibitors. The protein content of cell lysates was measured by a colorimetric assay (Bio-Rad); cell lysates were electrophoresed in 10% SDS-PAGE and transferred onto a PVDF filter. The membrane was blocked with 5% milk and probed with 1 μg/ml of an anti-uPAR monoclonal antibody or anti-CXCR4 polyclonal antibodies. Finally, washed filters were incubated with horseradish peroxidase-conjugated secondary antibodies and bands detected by ECL.

### Real-time RT-PCR analysis

Cells were lysed in QIAZOL Reagent and total RNA was purified according to the manufacturer’s instructions. Real-time quantification of miRs was performed using the TaqMan MicroRNA Assay (Applied Biosystems, Foster City, CA, USA) according to the manufacturer’s instructions. RNU6 was used as endogenous control for miRNA expression studies; miRs with a C_T_>35 were treated as undetected.

To quantify uPAR-mRNA and CXCR4-mRNA, 1 μg of total RNA was reverse transcribed using the QuantiTect Reverse Transcription kit; 1 μl of a 1:10 dilution of reverse transcription reaction was analysed by real-time PCR with a Biorad IQ5 thermocycler, using IQ™SYBR Green Supermix for qPCR kit. The levels of specific mRNAs were normalized to the internal glyceraldeyde-3-phosphate dehydrogenase (GAPDH) mRNA. Primers, designed using Primer3 software (http://frodo.wi.mit.edu/cgi-bin/primer3/primer3_www.cgi) and used at 0.25 μM were as follows: for uPAR amplification, forward primer 5′-CTGGAGCTGGTGGAGAAAAG-3′ and reverse primer 5′-CATGTCTGATGAGCCACAGG-3′; for CXCR4 amplification, forward primer 5′-CTCAGACCACCGGTCTCTTC-3′ and reverse primer 5′-ATAGTCCCCTGAGCCCATTT-3′; for GAPDH amplification, forward primer 5′-GAAGGTGAAGGTCGGAGTC-3′ and reverse primer 5′-GAAGATGGTGATGGGATTTC-3′. The relative level of expression was calculated with the formula 2^−ΔΔct^.

### Cell migration and invasion assay

Migration of THP-1 cells, transfected with precursors of selected or control miRs, was performed in Boyden chambers, using uncoated 5 μm pore size PVPF polycarbonate filters. After 48 hrs from transfection, 2 × 10^5^ cells were loaded in the upper chamber in serum-free medium; 5 nM ATF, 100 nM SDF1 or 10% FBS-DMEM were added in the lower chamber as chemoattractant. Cells were allowed to migrate for 2 hrs at 37°C, 5% CO_2_. In invasion assays, 8 μm pore size filters were coated with 25 μg Matrigel (BD, Bedford, MA, USA) and cells were allowed to invade for 18 hrs at 37°C, 5% CO_2_. Then, the cells on the lower surface of the filter were fixed in ethanol, stained with haematoxylin, and counted at 20× magnification (10 random fields/filter).

### Cell proliferation assay

THP-1 cells were transfected with precursors of selected or control miRs. After 0, 24 or 48 hrs, cells from each transfection were diluted in 300 μl and distributed in three wells of 96-well plates. Cells were incubated for 1 hr at 37°C, 5% CO_2_, and then 20 μl/well of CellTiter 96 AQueous One Solution Reagent was added. After incubation for 4 hrs at 37°C, 5% CO_2_, the absorbance was determined by an ELISA reader (Bio-Rad) at a wavelength of 490 nm.

### Statistical analysis

Differences between groups were evaluated by the Student’s t-test using PRISM software (GraphPad, San Diego, CA). *P* *≤* 0.05 was considered statistically significant.

## Results

### Identification of miRs regulating uPAR and CXCR4 expression

We hypothesized that uPAR and CXCR4 expression could be co-regulated by same miRs in AML. Since both uPAR and CXCR4 are up-regulated in leukaemias, we focused on miRs endowed with oncosuppressor activity and involved in CD34^+^ HSCs mobilization and/or expressed in leukaemias.

On this basis, we firstly selected miR-146a, which has been previously reported to target and regulate the expression of CXCR4 37, and, thus, could represent a positive control in our experiments. miR-146a is involved in haematopoiesis 38, in haematological malignancies 38,39 and acts as a tumour suppressor 40. We further selected two miRs, miR-335 and miR-622, predicted *in silico,* by TargetScan and miRanda algorithms, to target uPAR-3′UTR and CXCR4-3′UTR, respectively. These miRs are expressed in mobilized CD34^+^HSCs and/or in leukaemia 41,42 and are reported to act as tumour suppressors 43,44. We then validated predicted targets of miR-335 and miR-622, and investigated whether miR-146a and miR-622 were also able to target uPAR-3′UTR and miR-335 could also target CXCR4-3′UTR.

**Figure 1:**
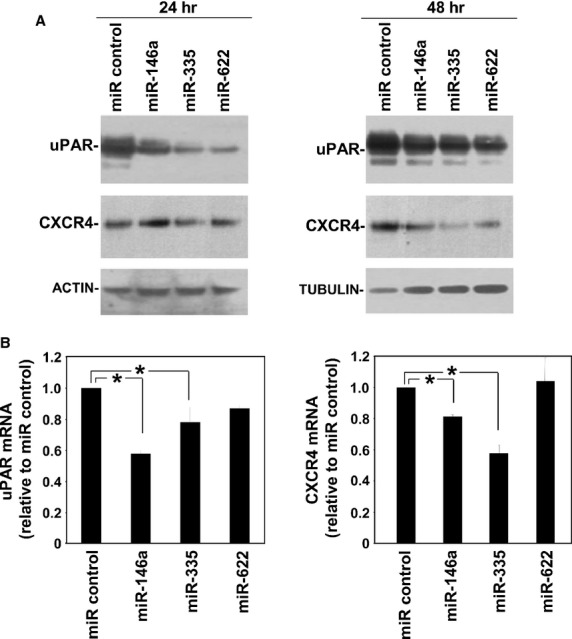
miR-146a, miR-335 and miR-622 impair uPAR and CXCR4 expression in uPAR/CXCR4-positive cells. (A) Hela cells were transfected with the synthetic precursors of miR-146a, miR-335, miR-622 or of a non-targeting control miR; after 24 and 48 hrs cells were lysed for Western blot analysis with uPAR- or CXCR4-specific antibodies; filters were reprobed with mouse anti-tubulin or rabbit anti-actin antibodies for loading control. (B) Hela cells were transfected with the synthetic precursors of miR-146a, miR-335, miR-622 or of a non-targeting control miR; then, cells were lysed with Qiazol for total RNA extraction and quantitative RT-PCR analysis with uPAR- and CXCR4-specific primers. uPAR and CXCR4 values were normalized to the GAPDH internal control. Results are expressed as ratio of uPAR- or CXCR4-mRNA levels in cells transfected with specific miRs over uPAR- and CXCR4- mRNA levels in cells transfected with control miR. Values are the mean ± SD of 3 independent experiments performed in triplicate. (*) *P* ≤ 0.05 as determined by the Student’s *t*-test.

To assess whether selected miRs were able to affect uPAR/CXCR4 expression, synthetic precursors of miR-146a, miR-335, miR-622 or of a control miR were transfected in HeLa recipient cells, which constitutively express both uPAR and CXCR4 5,45; cells were harvested and lysed at the indicated times. Evaluation of uPAR and CXCR4 expression by Western blot analysis with specific antibodies showed that all three selected miRs efficiently down-regulated both uPAR and CXCR4 expression at protein level, even if after different times of transfection, 24 hrs for uPAR and 48 hrs for CXCR4 (Fig.1A). Since miRs can regulate gene expression by inhibiting the translation of their target mRNAs and, often, inducing their degradation, total RNA was isolated from miR-transfected Hela cells and uPAR and CXCR4 mRNAs were quantized by qRT-PCR. The level of uPAR and CXCR4 transcripts in Hela cells transfected with miR-146a or miR-335 significantly decreased as compared to the control, whereas, miR-622 overexpression did not cause a significant reduction of uPAR and CXCR4 mRNAs (Fig.1B).

Similar results were obtained with another cell line expressing both uPAR and CXCR4, the prostate carcinoma PC3 cell line (not shown).

These results indicate that miR-146a, miR-335 and miR-622 are able to regulate uPAR and CXCR4 expression in cells constitutively expressing both receptors.

### uPAR and CXCR4 are direct targets of selected miRs

**Figure 2:**
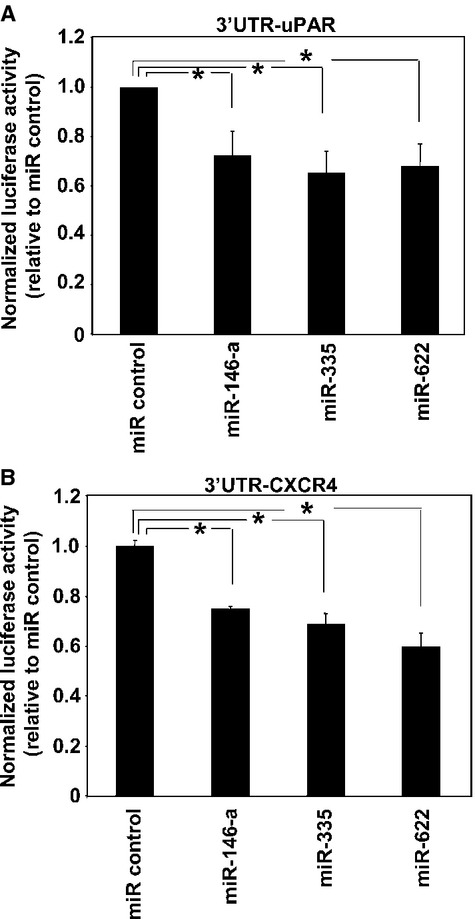
uPAR-mRNA and CXCR4-mRNA are direct targets of selected miRs. Full-length 3′untranslated region (UTR) of uPAR or CXCR4 mRNAs were cloned into the *firefly* luciferase-expressing pGL3 vector, just downstream of the luciferase stop codon. The resulting pGL-3′UTR/uPAR (A) or pGL-3′UTR/CXCR4 (B) constructs and the pRLSV40 vector, containing the *Renilla*-luciferase gene, were transiently co-transfected in Hela cells together with the synthetic precursors of a control non-targeting RNA (miR control) or with the synthetic precursors of indicated miRs. The relative *Firefly* luciferase activity was assayed 24 hrs after transfection and normalized to the internal control *Renilla*-luciferase. Then, values obtained in cells co-transfected with indicated miRs were expressed as ratio with values obtained in cells co-transfected with the control miR. Values are the mean ± SD of three experiments performed in triplicate. (*) *P* ≤ 0.05 as determined by the Student’s *t*-test.

To assess whether miR-146a, miR-335 and miR-622 directly target uPAR and CXCR4 mRNAs, 3′UTR sequence of uPAR and CXCR4 mRNAs was amplified from human genomic DNA and cloned into the *firefly* luciferase-expressing pGL3 vector, just downstream of the luciferase stop codon. The resulting pGL-3′UTR/uPAR or pGL-3′UTR/CXCR4 constructs and the pRLSV40 vector, containing the *Renilla*-luciferase gene (to normalize for transfection differences), were transiently co-transfected in Hela cells together with the synthetic precursor of a non-targeting RNA control miR or with the synthetic precursors of each selected miR. If selected miRs were able to directly bind the 3′UTR of uPAR and/or CXCR4, they should lower luciferase activity as compared to cells transfected with the control miR. Indeed, all selected miRs significantly reduced *firefly* luciferase activity, normalized to *Renilla*-luciferase activity, as compared to miR control, both in cells transfected with pGL-3′UTR/uPAR (Fig.2A) and in cells transfected with pGL-3′UTR/CXCR4 (Fig.2B).

Taken together, these results indicate that the analysed miRs directly target the 3′UTR of uPAR and CXCR4, thus being able to interfere with the expression of the corresponding mRNAs.

### Increased uPAR/CXCR4 expression is associated with decreased levels of selected miRs in leukaemia cell lines

We then evaluated the expression of selected miRs and of their identified targets, uPAR and CXCR4, in leukaemia cell lines. In fact, uPAR and CXCR4 expression in AML varies according to the FAB subtype (highest expression in M5 and lowest in M0) 21,22,25, thus leukaemia cells seemed a suitable system to validate the expression and function of identified miRs.

We focused on leukaemia cell lines derived from different AML subtypes and corresponding to different stages of differentiation; in particular, KG1 (M0/M1), THP-1 and U937 pro-monocytic (M5) cell lines were analysed.

**Figure 3:**
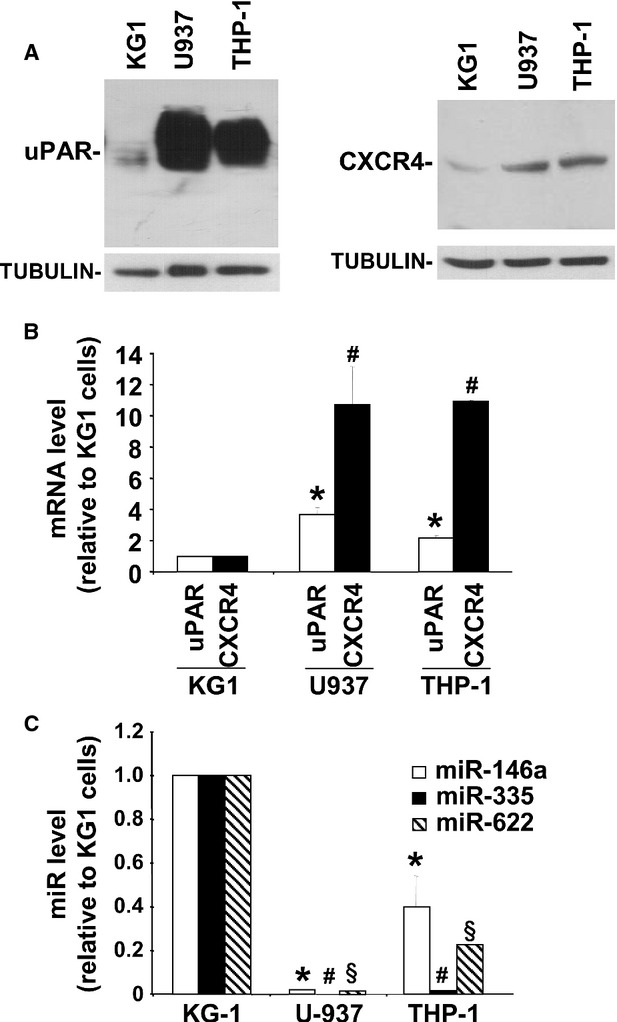
Increased uPAR/CXCR4 expression is associated with decreased levels of selected miRs in leukaemia cell lines. (A) Cell lysates from indicated leukaemia cell lines were analysed by Western blot with uPAR- or CXCR4-specific antibodies; filters where then re-probed with mouse anti-tubulin antibodies for loading control. (B) Cells from indicated leukaemia cell lines were lysed in Qiazol; uPAR- and CXCR4-mRNA levels were quantified by qPCR and normalized to the corresponding GAPDH mRNA levels. Values are the ratio between the levels of uPAR- or CXCR4-mRNA in U937 or THP-1 cells and their levels in KG1 cells. Values represent the mean ± SD of three separate experiments. (*) *P* ≤ 0.05 for uPAR-mRNA variation in U937 or THP-1 cells *versus* KG1 cells; (#) *P* ≤ 0.05 for CXCR4-mRNA variation in U937 or THP-1 cells *versus* KG1 cells; *P* determined by the Student’s *t*-test. (C) Cells were lysed in Qiazol; levels of miR-146a, miR-335 and miR-622 were quantized by quantitative Taqman RT-PCR and normalized to the U6 RNA internal control. Values are expressed as ratio between the levels of indicated miRs in THP-1 or U937 cells and their levels in KG1 cells. Values represent the mean ± SD of three separate experiments. (*) *P* ≤ 0.05 for miR-146a variation in U937 or THP-1 cells *versus* KG1 cells; (#) *P* ≤ 0.05 for miR-335 variation in U937 or THP-1 cells *versus* KG1 cells; (§) *P* ≤ 0.05 for miR-622 variation in U937 or THP-1 cells *versus* KG1 cells; *P* determined by the Student’s *t*-test.

We first evaluated uPAR and CXCR4 expression by Western blot with uPAR and CXCR4 specific antibodies (Fig.3A) and by quantitative real-time PCR analysis (qRT-PCR) with uPAR or CXCR4 specific primers (Fig.3B), showing that all three cell lines express uPAR and CXCR4 and that the expression of both molecules increases in THP-1 and U937 cells as compared to KG1 cells, at protein and mRNA level (Fig.3A and B).

Then, the expression of selected miRs was analysed by qRT-PCR, showing that miR-146a, miR-335 and miR-622 are expressed in leukaemia cells and that their levels decrease in THP-1 and U937 cells as compared to KG1 cells (Fig.3C).

Since miRs mediate post-transcriptional gene silencing by translational inhibition, the inverse relationship between the levels of selected miRs and the levels of uPAR/CXCR4 expression, observed in KG1 and THP-1 and U937 cells, suggested that miR-146a, miR-335 and miR-622 could regulate uPAR and CXCR4 expression in leukaemia cells.

### miR-146a, miR-335 and miR-622 regulate uPAR and CXCR4 expression in leukaemia cell lines

**Figure 4:**
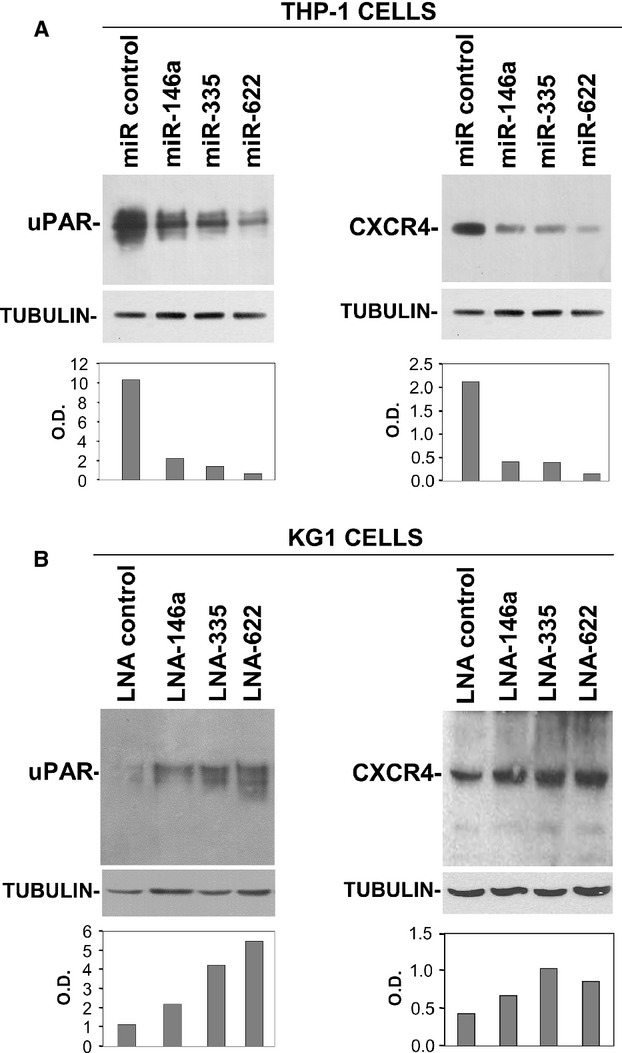
miR-146a, miR-335 and miR-622 regulate uPAR and CXCR4 expression in leukaemia cells. (A) THP-1 cells were transiently transfected with the synthetic precursors of miR-146a, miR-335, miR-622 or of a control miR; after 48 hrs, cells were lysed for Western blot analysis with uPAR- or CXCR4-specific antibodies. Filters were reprobed with a mouse anti-tubulin antibody for loading control. The graphs show the O.D. obtained by densitometric scanning of uPAR and CXCR4 bands normalized to the O.D. of corresponding tubulin. (B) KG1 cells were transiently transfected with LNAs specific for miR-146a, miR-335, miR-622 or a control LNA; after 24 hrs cells were lysed for Western blot analysis with uPAR- or CXCR4-specific antibodies. Filters were reprobed with a mouse anti-tubulin antibody for loading control. The graphs show the O.D. obtained by densitometric scanning of uPAR and CXCR4 bands normalized to the O.D. of corresponding tubulin.

To confirm that selected miRs are able to inhibit uPAR and CXCR4 expression in AML cell lines, we evaluated their effect on uPAR/CXCR4 expression in the pro-monocytic THP-1 cells, which showed lower miR levels and higher uPAR/CXCR4 expression as compared to KG1 cells (Fig.3). THP-1 cells were transiently transfected with the synthetic precursors of miR-146a, miR-335, miR-622 or of a control miR; after 48 hrs, transfected cells were lysed and cell lysates were analysed by Western blot with uPAR- and CXCR4- specific antibodies. Western blot analysis showed that all three miRs reduced the expression of both uPAR and CXCR4 as compared to the control miR (Fig.4A).

The activity of specific miRs can be impaired by specific miR inhibitors as locked nucleic acid (LNA)– oligonucleotides (ONs) 46. To investigate whether uPAR/CXCR4 expression in AML cell lines could be regulated by endogenously expressed selected miRs, LNAs specific for miR-146a, miR-335 and miR-622 or a control LNA were transfected in KG1 cells, which showed higher miR levels and lower uPAR/CXCR4 expression as compared to pro-monocytic cells (Fig.3). Then, transfected cells were analysed by Western blot with uPAR- and CXCR4-specific antibodies. Western blot analysis showed that all three LNA-ONs miR inhibitors increased the expression of both uPAR and CXCR4 respect to the control LNA-ON (Fig.4B).

These results indicate that both overexpressed and endogenous miR-146a, miR-335 and miR-622 regulate uPAR and CXCR4 expression in AML cell lines.

### miR-146a, miR-335 and miR-622 regulate THP-1 cell migration and invasion

uPAR expression regulates cell migration by interacting with cell surface molecules and CXCR4 plays an important role in the regulation of AML cell migration 2,19,25. We first evaluated whether selected miRs, which are able to down-regulate uPAR/CXCR4 expression, can influence cell migration.

**Figure 5:**
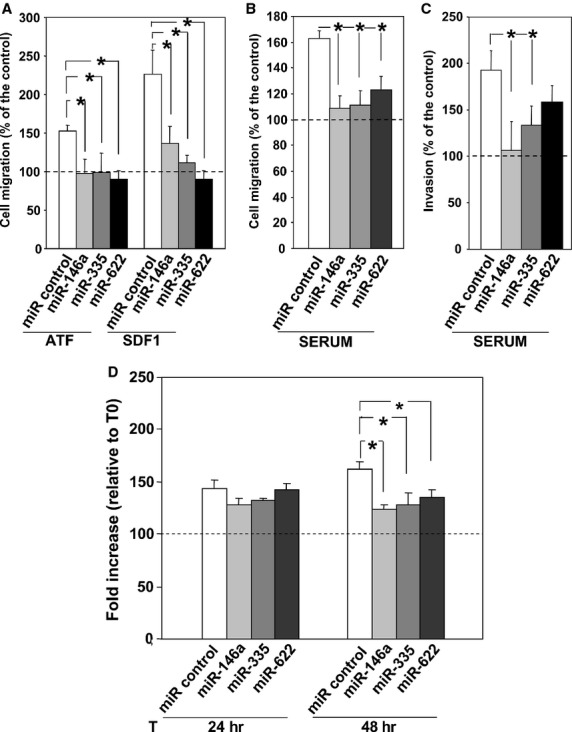
miR-146a, miR-335 and miR-622 regulate THP-1 cell migration, invasion and proliferation. (A–C) THP-1 cells were transiently transfected with the synthetic precursors of miR-146a, miR-335, miR-622 or of a control miR. Then, cells were loaded in Boyden chamber on uncoated filters (A and B) or Matrigel coated filters (C). Then, cells were allowed to migrate towards the uPA aminoterminal fragment (ATF) or the stroma derived factor 1 (SDF1) (A) or towards 10% serum (FBS) (B and C). Migrated cells were fixed, stained with haematoxylin, and counted. Results of migration assays are expressed as percentage of cells migrated towards chemoattractants over the cells migrated without chemoattractants; 100% values represent cell migration in the absence of chemoattractants. The values are the mean ± SEM of three experiments performed in triplicate. (*) *P* ≤ 0.05, as determined by the Student’s *t*-test. (D) THP-1 cells were transfected with the synthetic precursors of miR-146a, miR-335, miR-622 or of a control miR. Cells, harvested at indicated times, were loaded in 96-well plates and incubated with 20 μl/well of CellTiter 96 AQueous One Solution Reagent for 4 hrs at 37°C, 5% CO_2_. Then, the absorbance was determined by an ELISA reader (Bio-Rad) at a wavelength of 490 nm. Fold increase represents the ratio between the OD at indicated time points and OD at time 0. The values are the mean of three experiments performed in triplicate. (*) *P* ≤ 0.05, as determined by the Student’s *t*-test.

THP-1 cells were transfected with precursors of miR-146a, miR-335, miR-622 or of a control miR and, after 48 hrs, were loaded in Boyden chambers, to evaluate their migration towards the aminoterminal fragment of uPA (ATF) or SDF1, respectively, uPAR and CXCR4 ligands. THP-1 cells, transfected with the control miR, efficiently migrated towards both ATF and SDF1; overexpression of all three selected miRs significantly impaired THP-1 cell ability to migrate towards both chemoattractants (Fig.5A).

Since we recently showed that uPAR silencing by specific siRNAs abrogates directional cell migration, independently on the specific chemoattractant 2, migration assays towards serum were also performed, showing inhibition of migration in THP-1 cells transfected with selected miRs (Fig.5B). Expression of selected miRs also impaired THP-1 cell capability to invade Matrigel, even if miR-622 did not inhibit in a significant manner (Fig.5C).

Thus, miR-146a, miR-335 and miR-622 negatively regulate THP-1 cell migration and invasion; this effect could be attributed, at least partially, to their capability to impair uPAR and CXCR4 expression. However, since miRs are multi-target molecules, it is possible that selected miRs down-regulate also the expression of other molecules crucial in directional cell migration.

### miR-146a, miR-335 and miR-622 influence THP-1 cell proliferation

uPAR expression is able to regulate also cell proliferation by interacting with cell surface molecules 47. CXCR4 also regulates AML cell proliferation and retention within the BM 24. We then investigated whether selected miRs, which are able to down-regulate uPAR/CXCR4 expression, can influence also cell proliferation.

THP-1 cells were transfected with precursors of miR-146a, miR-335, miR-622 or of a control miR and, after 0, 24 and 48 hrs, cell number was evaluated by a colorimetric assay. The fold increase of transfected THP-1 cells was similar 24 hrs from transfection, whereas, 48 hrs after transfection, the fold increase of cells transfected with selected miRs was significantly lower as compared to that of control cells (Fig.5D).

Apoptosis induced by serum deprivation was also evaluated in miR-transfected cells, without observing any influence of selected miRs on THP-1 cell survival (data not shown).

Thus, miR-146a, miR-335 and miR-622 seem to affect also proliferation of AML cells; since uPAR and CXCR4 are both involved in the regulation of cell proliferation, it is possible that their miR-mediated down-regulation may contribute to this effect.

### Analysis of uPAR/CXCR4 expression and selected miRs in AML blasts

We finally investigated expression of uPAR and CXCR4 and of selected miRs in blasts obtained from 10 AML patients, and compared them to CD34^+^ HSCs obtained from three healthy donors.

**Figure 6:**
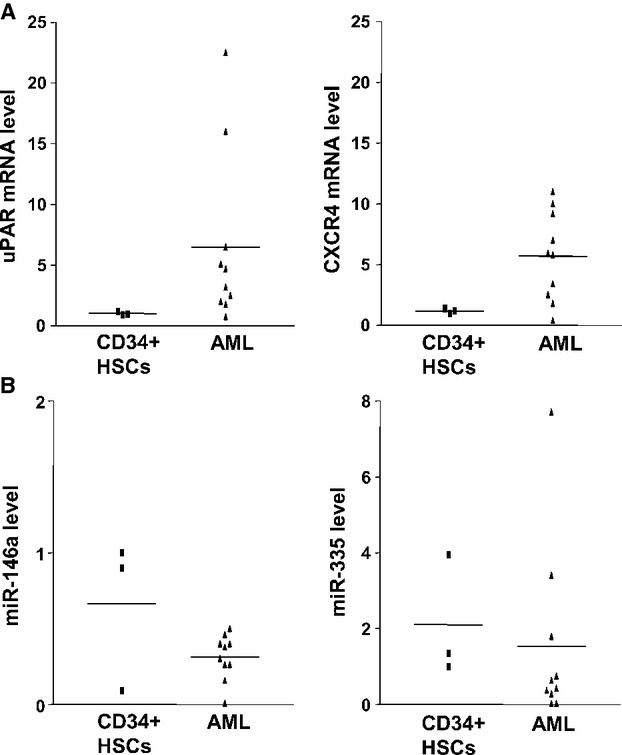
uPAR/CXCR4 expression and levels of selected miRs in AML samples. (A) CD34^+^ hematopoietic stem cells (HSCs) from 3 healthy donors and blasts from 10 patients affected by acute myeloid leukaemia (AML) were lysed in Qiazol and analysed by quantitative RT-PCR with uPAR or CXCR4 specific primers. uPAR and CXCR4 values were normalized to GAPDH. (B) Levels of mature miR-146a, and miR-335 in normal HSCs and AML blasts from same donors of panel A were analysed by quantitative Taqman RT-PCR. MiR levels were normalized to U6 internal control. Data represent the average ± SD of three independent experiments performed in triplicate.

The levels of uPAR and CXCR4 expression in AML blasts, analysed by qRT-PCR, were quite heterogeneous; however, the mean of both uPAR and CXCR4 expression was higher as compared to their expression in normal CD34^+^ HSCs (Fig.6A).

Then, miR levels were evaluated by qRT-PCR in same AML blasts and normal CD34^+^ HSCs (Fig.6B). The mean of miR-146a levels was significantly lower in AML blasts as compared to its level in normal CD34^+^ HSCs (*P* < 0.05), showing an inverse relationship between its expression and uPAR and CXCR4 expression. This result confirms that miR-146a down-regulation may up-regulate uPAR/CXCR4 expression in AML also *in vivo*, as observed in leukaemia cell lines. The mean of miR-335 also decreased in AML blasts as compared to normal CD34^+^ HSCs, although the difference was not statistically significant. By contrast, miR-622 was undetectable.

These data indicate that miR-146a and miR-335 are expressed in normal CD34^+^ HSCs and may be involved in the regulation of uPAR/CXCR4 expression in AML blasts *in vivo*.

## Discussion

uPAR and CXCR4 expression is strongly up-regulated and represents a negative prognostic factor in various cancers; in fact, both receptors are involved in tumour progression, angiogenesis and metastasis formation 11,19,20,48. uPAR and CXCR4 co-expression predicts worse prognosis in small cell lung cancer patients 49, characterizes mesenchymal circulating tumour cells from breast cancer patients with lymph nodes involvement 50, increases in hypoxic cells sorted from cervical and lymph nodal xenograft tumours 51, is down-regulated by antimetastatic COX-2 inhibitors 52. Interestingly, CXCR4 stimulation induces up-regulation of uPAR expression in breast cancer cell lines 53. The levels of uPAR and CXCR4 increase also in haematological malignancies; their expression in acute myeloid leukaemia (AML) varies according to the subtype, with lower expression in undifferentiated (M0) and higher expression in myelomonocytic (M4/5) AML 21,22,25. Both receptors seem involved in the proliferation/survival of HSCs and in their trafficking from/to the BM 10,12–15. We recently showed a functional cross-talk between uPAR and CXCR4; in fact, uPAR expression regulates SDF1-induced cell migration on different components of the ECM, impairing migration on collagen and promoting it on VN 9. Thus, we hypothesized that the expression of both receptors could be co-regulated, thus allowing their cross-talk to realize a common end. A probable way to have a coordinated expression of different molecules is to be targeted by same miRs. In fact, miRs are multi-target molecules which can simultaneously regulate the expression of various factors. Indeed, miRs are involved in the control of differentiation and activity of hematopoietic cells and are frequently deregulated in human malignancies 29–35. A direct miR-mediated regulation of uPAR or CXCR4 expression in leukaemias has been scarcely investigated. Thus, we selected three miRs to investigate a possible miR-mediated co-regulation of uPAR and CXCR4 expression. We focused on miR-146a, which can directly target CXCR4 and is strongly involved both in myelopoiesis and in acute myeloid leukaemia 37–40; interestingly, in melanoma cells, miR-146a targets the heterogeneous nuclear ribonucleoprotein C1/C2 (hnRNPC), a mRNA-binding protein which, in turn, is able to regulate the expression of uPA and its receptor 54,55. We also selected miR-335 and miR-622, which are predicted to target uPAR- or CXCR4- 3′UTR and are expressed in HSC mobilization and/or leukaemia 41–43.

We firstly evaluated the capability of selected miRs to regulate uPAR and CXCR4 expression in cells expressing both receptors, showing that all of them directly target both uPAR and CXCR4 mRNAs. Then, we evaluated their expression in leukaemia cell lines characterized by a different uPAR/CXCR4 expression, low in KG1 cells, derived from an M0 subtype of AML, and high in pro-monocytic THP-1 and U937 cells, showing an inverse expression of selected miRs, consistent with the hypothesis that these miRs directly regulate uPAR/CXCR4 expression. Furthermore, overexpression of all selected miRs lowered uPAR/CXCR4 expression in THP-1 cells and, viceversa, the specific inhibition of all three miRs increased uPAR/CXCR4 expression in KG1 cells, confirming a functional role of selected miRs in leukaemia cell lines.

To explore the functional meaning of our observations, we evaluated the effect of selected miRs on migration of THP-1, the cell line expressing high levels of uPAR and CXCR4, towards the ligands of both receptors. Then, since we recently showed that uPAR expression controls cell migration independently of the specific chemoattractant, we also assayed cell migration towards serum. Chemotaxis assays showed that all three miRs significantly reduced directional migration of THP-1 cells; these results may be partly attributed to uPAR and CXCR4 down-regulation, without excluding the contribute of other targets possibly required for cell migration. Furthermore, expression of selected miRs impaired Matrigel invasion, even if miR-622-dependent inhibition was not statistically significant.

Similar results were obtained in proliferation assays; since both uPAR and CXCR4 can transduce proliferation signals 19,47, also this effect may be attributed to their down-regulation without excluding the involvement of other targets of selected miRs.

Finally, we showed expression of miR-146a and miR-335 also *in vivo*, in normal CD34^+^ HSCs and in AML blasts. miR-146a levels significantly decreased and the expression of its targets, uPAR and CXCR4, increased in AML blasts, as compared to normal HSCs; thus, it is possible to speculate that this specific miR may regulate uPAR/CXCR4 also *in vivo*. MiR-335 level also decreased but not in a statistically significant manner; that could be related to reports showing that miR-335 is up-regulated in a subset of high risk AMLs associated with specific mutations 56,57.

A possible correlation between uPAR expression and the clinical features of acute leukaemias was previously observed in AML patients; a high uPAR expression, irrespective of the FAB category, showed a greater tendency to cutaneous and tissue infiltration, together with a higher leukocyte count, as compared to AML patients whose blasts were uPAR negative or dimly positive 21,22. On the other hand, CXCR4 is an important player in the cross-talk between leukaemia cells and the BM microenvironment; CXCR4 expression is associated with poor prognosis in AML patients since its expression and binding to SDF1, produced by BM cells, is critical for the survival and retention of AML cells within the BM. In fact, *in vivo*, CXCR4 antagonists were found to induce the mobilization of AML cells and progenitor cells into the circulation and enhance anti-leukaemic effects of chemotherapy. The hypothesis that CXCR4 contributes to the resistance of AML cells to signal transduction inhibitor- and chemotherapy-induced apoptosis is currently being tested in a series of Phase I/II studies in humans 58.

All together, our results indicate that uPAR and CXCR4, which are able to cross-talk each other, to regulate trafficking and proliferation/survival of HSCs and which are strongly involved in AML, are direct targets of miR-146a, miR-335 and miR-622. In particular, miR-146a, whose deletion in mouse models leads to myeloproliferative disorders 38–40, may target uPAR/CXCR4 also in human AML and, thus, might represent a useful tool in therapeutical approaches.

## References

[b1] Ragno P (2006). The urokinase receptor: a ligand or a receptor? Story of a sociable molecule. Cell Mol Life Sci.

[b2] Gorrasi A, Li Santi A, Amodio G (2014). The urokinase receptor takes control of cell migration by recruiting integrins and FPR1 on the cell surface. PLoS ONE.

[b3] Montuori N, Ragno P (2009). Multiple activities of a multifaceted receptor: roles of cleaved and soluble uPAR. Front Biosci.

[b4] Alfano D, Ragno P, Stoppelli MP (2012). RhoB regulates uPAR signalling. J Cell Sci.

[b5] Montuori N, Cosimato V, Rinaldi L (2013). uPAR regulates pericellular proteolysis through a mechanism involving integrins and fMLF-receptors. Thromb Haemost.

[b6] Smith HW, Marshall CJ (2010). Regulation of cell signalling by uPAR. Nat Rev Mol Cell Biol.

[b7] Montuori N, Visconte V, Rossi G (2005). Soluble and cleaved forms of the urokinase-receptor: degradation products or active molecules?. Thromb Haemost.

[b8] Resnati M, Pallavicini I, Wang JM (2002). The fibrinolytic receptor for urokinase activates the G protein-coupled chemotactic receptor FPRL1/LXA4R. Proc Natl Acad Sci USA.

[b9] Montuori N, Bifulco K, Carriero MV (2011). The cross-talk between the urokinase receptor and fMLP receptors regulates the activity of the CXCR4 chemokine receptor. Cell Mol Life Sci.

[b10] Selleri C, Montuori N, Ricci P (2005). Involvement of the urokinase-type plasminogen activator receptor in hematopoietic stem cell mobilization. Blood.

[b11] Furusato B, Mohamed A, Uhlen M (2010). CXCR4 and cancer. Pathol Int.

[b12] Motabi IH, DiPersio JF (2012). Advances in stem cell mobilization. Blood Rev.

[b13] Selleri C, Montuori N, Ricci P (2006). *In vivo* activity of the cleaved form of soluble urokinase receptor: a new hematopoietic stem/progenitor cell mobilizer. Cancer Res.

[b14] Wysoczynski M, Reca R, Ratajczak J (2005). Incorporation of CXCR4 into membrane lipid rafts primes homing-related responses of hematopoietic stem/progenitor cells to an SDF-1 gradient. Blood.

[b15] Tjwa M, Sidenius N, Moura R (2009). Membrane-anchored uPAR regulates the proliferation, marrow pool size, engraftment, and mobilization of mouse hematopoietic stem/progenitor cells. J Clin Invest.

[b16] Vallabhaneni KC, Tkachuk S, Kiyan Y (2011). Urokinase receptor mediates mobilization, migration, and differentiation of mesenchymal stem cells. Cardiovasc Res.

[b17] Kucia M, Reca R, Miekus K (2005). Trafficking of normal stem cells and metastasis of cancer stem cells involve similar mechanisms: pivotal role of the SDF-1-CXCR4 axis. Stem Cells.

[b18] Schroeder MA, DiPersio JF (2012). Mobilization of hematopoietic stem and leukemia cells. J Leukoc Biol.

[b19] Beverly A, Teicher BA, Fricker SP (2010). CXCL12 (SDF-1)/CXCR4 pathway in cancer. Clin Cancer Res.

[b20] Noh H, Hong S, Huang S (2013). Role of urokinase receptor in tumor progression and development. Theranostics.

[b21] Bene MC, Castoldi G, Knapp W (2004). CD87 (urokinase-type plasminogen activator receptor), function and pathology in hematological disorders: a review. Leukemia.

[b22] Lanza F, Castoldi GL, Castagnari B (1998). Expression and functional role of urokinase-type plasminogen activator receptor in normal and acute leukaemic cells. Br J Haematol.

[b23] Burger JA, Burkle A (2007). The CXCR4 chemokine receptor in acute and chronic leukaemia: a marrow homing receptor and potential therapeutic target. Br J Haematol.

[b24] Burger JA, Peled A (2009). CXCR4 antagonists: targeting the microenvironment in leukemia and other cancers. Leukemia.

[b25] Mohle R, Schittenhelm M, Failenschmid C (2000). Functional response of leukaemic blasts to stromal cell-derived factor-1 correlates with preferential expression of the chemokine receptor CXCR4 in acute myelomonocytic and lymphoblastic leukaemia. Br J Haematol.

[b26] Montuori N, Rossi G, Ragno P (2002). Post-transcriptional regulation of gene expression in the plasminogen activation system. Biol Chem.

[b27] Bartel DP (2009). MicroRNAs: target recognition and regulatory functions. Cell.

[b28] Treiber T, Treiber N, Meister G (2012). Regulation of microRNA biogenesis and function. Thromb Haemost.

[b29] Bissels U, Bosio A, Wagner W (2012). MicroRNAs are shaping the hematopoietic landscape. Haematologica.

[b30] Havelange V, Garzon R (2010). MicroRNAs emerging key regulators of hematopoiesis. Am J Hematol.

[b31] Di Leva G, Garofalo M, Croce CM (2014). MicroRNAs in cancer. Annu Rev Pathol.

[b32] Garzon R, Marcucci G (2012). Potential of microRNAs for cancer diagnostics, prognostication and therapy. Curr Opin Oncol.

[b33] Havelange V, Garzon R, Croce CM (2009). MicroRNAs: new players in acute myeloid leukaemia. Br J Cancer.

[b34] Marcucci G, Mrozek K, Radmacher MD (2011). The prognostic and functional role of microRNAs in acute myeloid leukemia. Blood.

[b35] Wang Y, Li Z, He C (2010). MicroRNAs expression signatures are associated with lineage and survival in acute leukemias. Blood Cells Mol Dis.

[b36] Montuori N, Selleri C, Risitano AM (1999). Expression of the 67-kDa laminin receptor in acute myeloid leukemia cells mediates adhesion to laminin and is frequently associated with monocytic differentiation. Clin Cancer Res.

[b37] Labbaye C, Spinello I, Quaranta MT (2008). A three-step pathway comprising PLZF/miR-146a/CXCR4 controls megakaryopoiesis. Nat Cell Biol.

[b38] So AY, Zhao JL, Baltimore D (2013). The Yin and Yang of microRNAs: leukemia and immunity. Immunol Rev.

[b39] Hua Z, Chun W, Fang-Yuan C (2011). MicroRNA-146a and hemopoietic disorders. Int J Hematol.

[b40] Labbaye C, Testa U (2012). The emerging role of MIR-146A in the control of hematopoiesis, immune function and cancer. J Hematol Oncol.

[b41] Bryant A, Palma C, Jayaswal V (2012). miR-10a is aberrantly overexpressed in Nucleophosmin1 mutated acute myeloid leukaemia and its suppression induces cell death. Mol Cancer.

[b42] Donahue RE, Jin P, Bonifacino AC (2009). Plerixafor (AMD3100) and granulocyte colony-stimulating factor (G-CSF) mobilize different CD34^+^ cell populations based on global gene and microRNA expression signatures. Blood.

[b43] Han Z, Yang Q, Liu B (2012). MicroRNA-622 functions as a tumor suppressor by targeting K-Ras and enhancing the anticarcinogenic effect of resveratrol. Carcinogenesis.

[b44] Tavazoie SF, Alarcon C, Oskarsson T (2008). Endogenous human microRNAs that suppress breast cancer metastasis. Nature.

[b45] Harada S, Maeda Y (1999). Chemically induced infection of CD4-negative HeLa cells with HIV-1. Microbiol Immunol.

[b46] Stenvang J, Silahtaroglu AN, Lindow M (2008). The utility of LNA in microRNA-based cancer diagnostics and therapeutics. Semin Cancer Biol.

[b47] Alfano D, Franco P, Vocca I (2005). The urokinase plasminogen activator and its receptor: role in cell growth and apoptosis. Thromb Haemost.

[b48] Montuori N, Ragno P (2014). Role of uPA/uPAR in the modulation of angiogenesis. Chem Immunol Allergy.

[b49] Li Y, Shen Y, Miao Y (2014). Co-expression of uPAR and CXCR4 promotes tumor growth and metastasis in small cell lung cancer. Int J Clin Exp Pathol.

[b50] Markiewicz A, Książkiewicz M, Wełnicka-Jaśkiewicz M (2014). Mesenchymal phenotype of CTC-enriched blood fraction and lymph node metastasis formation potential. PLoS ONE.

[b51] Chaudary N, Hill RP (2009). Increased expression of metastasis-related genes in hypoxic cells sorted from cervical and lymph nodal xenograft tumors. Lab Invest.

[b52] Silva HC, Alves V, Nogueira LA (2012). Impairment of breast cancer cell invasion by COX-2-specific inhibitor xml: roles of CXCR4 and of uPA system. Med Oncol.

[b53] Serratì S, Margheri F, Fibbi G (2008). Endothelial cells and normal breast epithelial cells enhance invasion of breast carcinoma cells by CXCR-4-dependent up-regulation of urokinase-type plasminogen activator receptor (uPAR, CD87) expression. J Pathol.

[b54] Hwang SJ, Seol HJ, Park YM (2012). MicroRNA-146a suppresses metastatic activity in brain metastasis. Mol Cells.

[b55] Abba M, Patil N, Allgayer H (2014). MicroRNAs in the regulation of MMPs and metastasis. Cancers.

[b56] Marcucci G, Maharry K, Radmacher MD (2008). Prognostic significance of, and gene and microRNA expression signatures associated with, CEBPA mutations in cytogenetically normal acute myeloid leukemia with high-risk molecular features: a Cancer and Leukemia Group B Study. J Clin Oncol.

[b57] Shivarov V, Bullinger L (2014). Expression profiling of leukemia patients: key lessons and future directions. Exp Hematol.

[b58] Peled A, Tavor S (2013). Role of CXCR4 in the pathogenesis of acute myeloid leukemia. Theranostics.

